# Probing the Conformational States of a pH-Sensitive DNA Origami Zipper via Label-Free Electrochemical Methods

**DOI:** 10.1021/acs.langmuir.1c01110

**Published:** 2021-06-15

**Authors:** Paul Williamson, Heini Ijäs, Boxuan Shen, Damion K. Corrigan, Veikko Linko

**Affiliations:** † Department of Biomedical Engineering, University of Strathclyde, 40 George Street, Glasgow G1 1QE, United Kingdom; ‡ Biohybrid Materials, Department of Bioproducts and Biosystems, Aalto University, P.O. Box 16100, 00076 Aalto, Finland; § Nanoscience Center, Department of Biological and Environmental Science, University of Jyväskylä, P.O. Box 35, 40014 Jyväskylä, Finland; ∥ HYBER Centre, Department of Applied Physics, Aalto University, P.O. Box 15100, 00076 Aalto, Finland

## Abstract

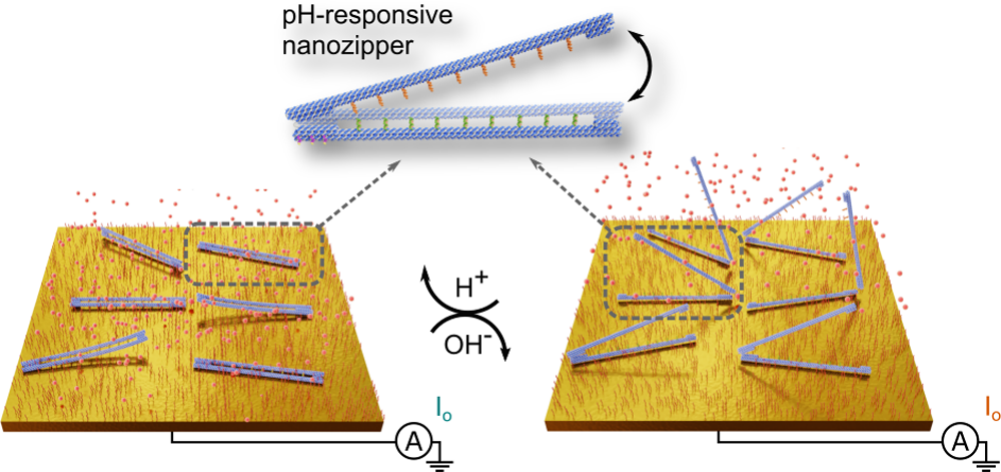

DNA origami structures represent an exciting class of materials for use in a wide range of biotechnological applications. This study reports the design, production, and characterization of a DNA origami “zipper” structure, which contains nine pH-responsive DNA locks. Each lock consists of two parts that are attached to the zipper’s opposite arms: a DNA hairpin and a single-stranded DNA that are able to form a DNA triplex through Hoogsteen base pairing. The sequences of the locks were selected in a way that the zipper adopted a closed configuration at pH 6.5 and an open state at pH 8.0 (transition p*K*_a_ 7.6). By adding thiol groups, it was possible to immobilize the zipper structure onto gold surfaces. The immobilization process was characterized electrochemically to confirm successful adsorption of the zipper. The open and closed states were then probed using differential pulse voltammetry and electrochemical impedance spectroscopy with solution-based redox agents. It was found that after immobilization, the open or closed state of the zipper in different pH regimes could be determined by electrochemical interrogation. These findings pave the way for development of DNA origami-based pH monitoring and other pH-responsive sensing and release strategies for zipper-functionalized gold surfaces.

## 1 Introduction

Electrochemical DNA biosensing may enable low cost, reliable, and specific detection of various known and emerging biomarkers associated with human disease. Introduction of ordered monolayers of single stranded DNA to an electrode by self-assembly techniques provides a method of capturing and detecting complementary target sequences of interest from solution. Applications of this sensing principle are far reaching, including the detection of bacterial nucleic acids associated with AMR,^1−3^ circulating tumor DNA sequences,^4,5^ and single nucleotide polymorphisms.^6,7^ Despite much promise in laboratories worldwide, translation into clinical or field settings has proved challenging. Known issues of sensor stability, signal drift, and performance in complex media are still to be overcome.^8,9^

Attempts to improve the sensitivity, specificity, and signal amplification of DNA biosensing have contributed to the introduction of ever more complex surface modifications. Increasing structural complexities of the sensing regions,^10−14^ tethering of redox active mediators to DNA to allow for a ratiometric approach,^15−17^ and translation to a microelectrode platform^18^ have all gone some way to improving sensor performance and reliability. However, many sensors are still limited by the success rate of self-assembly methods, their inherent variability in establishing an appropriate baseline signal, and corresponding signal drift.

Higher order DNA structures, such as DNA origami,^19,20^ have recently found a plethora of uses in various scientific areas^21,22^ ranging from super-resolution imaging^23^ to drug delivery.^24^ Equally, these structures may be integrated to outer circuitry and interfaces as pegboards, photonic and electronic elements, and switches.^25−31^ Therefore, they may provide a means of better managing packing densities, enhancing sensitivity by signal amplification, and introducing greater functionality to a sensor. Conformational switching is also possible in response to given environmental stimuli such as temperature gradients, strand displacement reactions, DNA–protein interactions, taking advantage of the photoactivated properties of the system, or more recently the local environmental pH.^32−37^ Switchable DNA origami structures have been used for constructing DNA origami sensors with optical readout, such as plasmonics^38^ and various fluorescence and surface-enhanced Raman scattering (SERS)-based methods.^39^ To our knowledge, the application of structures derived from DNA origami for use in electrochemical biosensing has been largely limited to static DNA constructs.^40−42^

**Figure 1:**
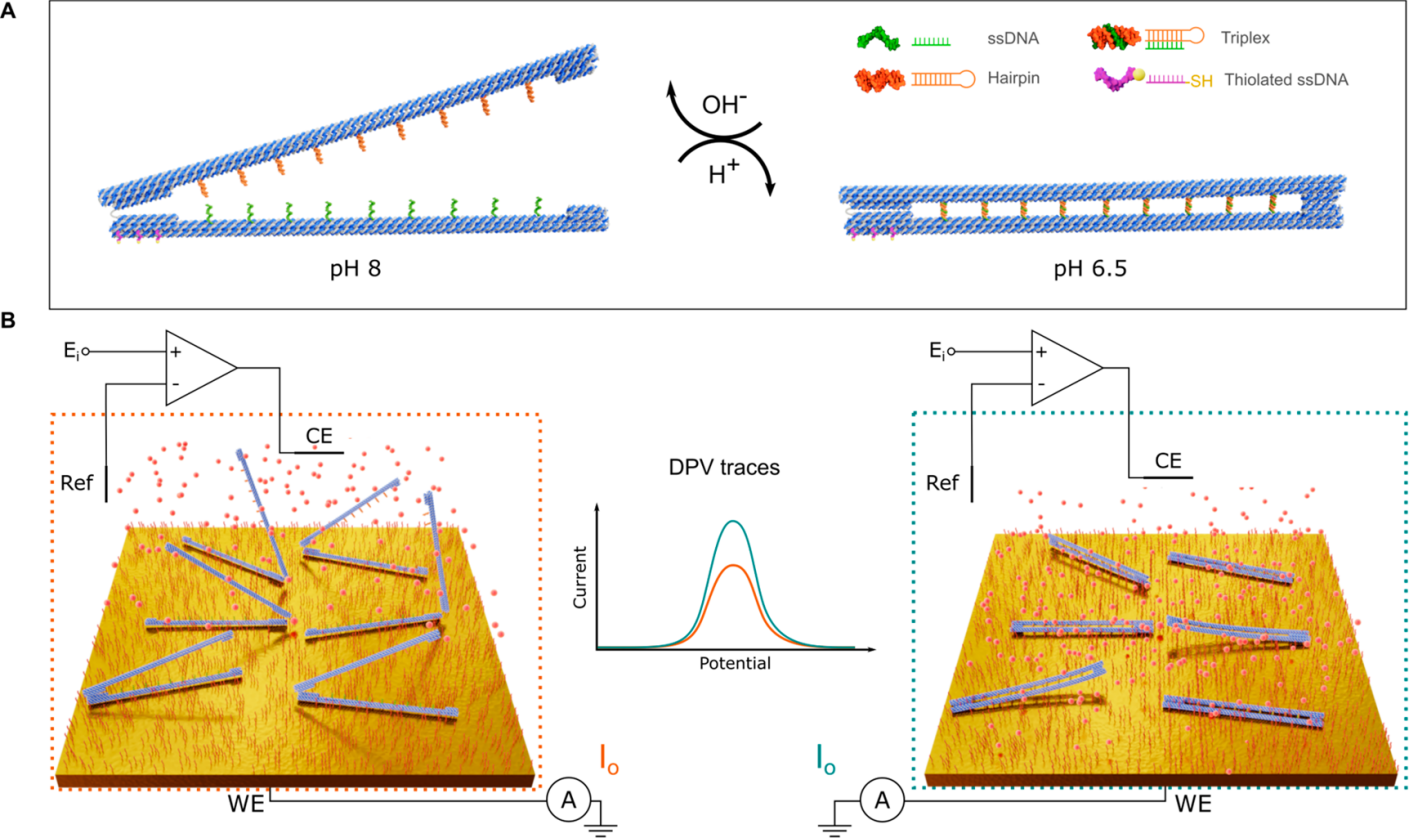
Schematics of the DNA origami zipper. (A) The conformational states of the zipper at pH 8 (left) and pH 6.5 (right). (B) The zippers are immobilized onto the gold electrode surface through thiol-modifications (purple strands in A). The opening and closing of the zipper modulate the average distance of the redox mediators (red spheres) from the electrode surface, thus resulting in a detectable current signal change in differential pulse voltammetry (DPV) traces. WE and CE denote the working electrode and the counter electrode, respectively.

Here we have employed an unlabeled switchable/dynamic DNA origami zipper device (Figure 1), which we aim to observe via electrochemical methods of differential pulse voltammetry (DPV) and electrochemical impedance spectroscopy (EIS). This is of immediate interest to future electrochemical biosensing applications for numerous reasons. First, the electrochemical driving of solution pH change by an applied potential through an electrode is well documented.^43^ These structures are also readily modifiable to harbor recognition sites for target oligonucleotides, capable of encapsulating or tethering a range of signaling molecules or for the loading of a desired cargo molecule for a site-specific release.^35^

## 2 Materials and Methods

### 2.1 DNA Origami Zipper Design and Assembly

#### 2.1.1 Materials

The 7560-nt single-stranded DNA scaffold for zipper assembly was purchased from Tilibit Nanosystems. The staple oligonucleotides, including the thiol-modified oligonucleotides for gold immobilization, were purchased from Integrated DNA Technologies. The 50× TAE buffer was purchased from VWR Chemicals, the agarose from Thermo Fisher Scientific, and the gel loading dye and ethidium bromide from Sigma-Aldrich. Deionized (DI) water of Milli-Q grade was used in all sample preparation and analysis steps.

#### 2.1.2 Design, Assembly, and Purification

The zipper DNA origami structure was designed on a honeycomb lattice with the caDNAno software version 2.2.0.^44^ The 3D solution structure and flexibility were predicted with the CanDo online software.^45,46^ The sequences of the pH-responsive DNA triplexes (pH locks) were designed according to the reported dependency of the acid dissociation constant (p*K*_a_) on the percentage of TAT base triplets in the triplex sequence (%TAT).^32,35,47^ The NUPACK online simulation tool^48^ was used to ensure a correct secondary structure formation and a sufficient melting temperature of the DNA hairpins in the pH locks.

Folding reactions of the DNA zipper contained the circular 7560-nt scaffold strand at 20 nM concentration and a set of 216 staple oligonucleotides (see Tables S1–S3 in Supporting Information) in a 9.2× molar excess to the scaffold in 1× folding buffer (FOB; 1× TAE and 15 mM MgCl_2_ at pH ∼ 8.3). The structures were folded by heating the mixture to 90 °C and cooling to 27 °C with the following thermal annealing program in a G-Storm G1 thermal cycler: (1) Cooling from 90 to 70 °C at a rate of −0.2 °C/8 s; (2) cooling from 70 to 60 °C at a rate of −0.1 °C/8 s; (3) cooling from 60 to 27 °C at a rate of −0.1 °C/2 min. The reactions were then cooled to 12 °C until the program was manually stopped. After folding, the structures were stored at 4 °C. The excess staple strands in the folding mixture were removed with polyethylene glycol (PEG) precipitation.^49^ The folding mixture was diluted with a factor of 1:4 with 1× FOB and mixed at a 1:1 ratio with PEG precipitation buffer (1× TAE, 505 mM NaCl, 15% (w/v) PEG8000). The mixture was centrifuged for 30 min at 14 000*g* at room temperature (RT), the supernatant was discarded, and the pellet was resuspended in the original volume of 1× FOB by incubating at RT overnight.

The concentration of the DNA origami samples was estimated with the Beer–Lambert law and sample absorbance at 260 nm (*A*_260_ = ε_260_ × *c* × *l*). The molar extinction coefficient at 260 nm for the zippers was estimated as ε_260_ = 10.7 × 10^7^ M^–1^ cm^–1^,^50^ according to the number of dsDNA (N_ds_) and ssDNA nucleotides (N_ss_) in the structures (*N*_ds_ = 14,820 and *N*_ss_ = 799 for both the active zippers and the open controls).

For studying the conformational state of the zippers in different pH media with AFM and AGE, the 1× FOB of PEG-purified zippers was exchanged for either 1× TAE buffer (pH 6.5 or pH 8.0) or 100 mM phosphate buffer (pH 6.5), each supplemented with 15 mM MgCl_2_ and 5 mM NaCl. The buffer exchange was carried out with spin-filtration using Amicon Ultra 0.5 mL spin-filters with a 100 kDa molecular weight cutoff (Merck Millipore). The 1× FOB was first exchanged for DI water with two rounds of spin-filtration (6000*g*, 10 min, RT). The samples in DI water were then mixed in a 1:1 ratio with buffers prepared at a 2× concentration to yield the desired final buffer concentration and incubated overnight at RT before analysis.

#### 2.1.3 Atomic Force Microscopy (AFM)

The AFM characterization of zipper origami in 1× TAE buffer and phosphate buffer at pH 6.5 and pH 8.0 was carried out by a Dimension Icon AFM (Bruker). For sample preparation, the zipper samples were first diluted 2–5 fold with corresponding buffers to obtain optimal densities on the surface. Then 10 μL of diluted sample was drop-casted on a freshly cleaved mica surface and incubated for 30 s followed by washing with 100 μL of DI water three times and drying with N_2_ gas flow. The images were captured in ScanAsyst Mode with ScanAsyst-Air probes at 1 Hz scanning speed with 512 × 512 resolution. Image analysis for obtaining statistics of the zipper opening angles was performed using the angle measurement tool in ImageJ2 version 1.51g.^51^ For AFM imaging of zippers on gold substrate, the gold surface was prepared by evaporating 2 nm Ti and 20 nm Au to a p-type silicon chip by physical vapor deposition (PVD). An 8 nM thiolated zipper solution in 1× TAE buffer at pH 8.0 was incubated on the Au surface for 25 s followed by washing with 100 μL of DI water three times and drying with N_2_ gas flow. The images were captured with the same protocol as the samples on mica.

#### 2.1.4 Agarose Gel Electrophoresis (AGE)

The electrophoretic mobility of the zippers after folding, PEG purification, and buffer exchange was characterized with AGE. Agarose gels containing 2% (w/v) agarose and 0.47 μg/mL ethidium bromide were prepared in a running buffer with 1× TAE and 11 mM MgCl_2_ at pH ∼ 8.3. DNA samples were loaded on the gel in 1× loading dye. The gels were run at a constant voltage of 90 V for 45 min on an ice bath and imaged under UV light with either a BioRad ChemiDoc MP or a BioRad GelDoc XR+ imaging system.

### 2.2 Electrochemistry

#### 2.2.1 Materials

Polycrystalline gold electrodes (PGEs) of 2 mm diameter were purchased from IJ Cambria Scientific Ltd. (Llanelli, UK). 3-Mercapto-1-propanol (MCP) was obtained from Sigma-Aldrich (Dorset, UK). All other chemicals required were purchased from Acros Organics (Thermo Fisher Scientific Ltd.) (Geel, Belgium).

#### 2.2.2 Electrode Polishing and Cleaning

Appropriate cleaning is required to achieve conformity in electrode surfaces and the removal of immobilized organics and contaminants. Stripping of organics was attained by immersion of the gold surfaces in Piranha (H_2_SO_4_ and H_2_O_2_ 3:1 (v/v)) for 20 min at RT. Surfaces were then mechanically polished to a near mirror finish via a series of decreasing alumina slurry diameters from 1 to 0.03 μm, on microcloths of varying roughness, with sonication in IPA between each polishing step. Electrochemical cleaning was then undertaken by repeated cyclic voltammetry in 0.1 M H_2_SO_4_, until a stable reduction peak was observed in the voltammogram.

#### 2.2.3 Buffer Preparation

Electrochemical observations of DNA zipper conformation require repeat measurements, across a range of buffer pHs previously shown to induce either a closed or open state.^35^ Two buffering systems (in Table 1) across a pH range of 6.5 to 8, were employed in this work.

**Table 1:** Buffer Systems for the Determination of DNA Zipper Conformation

buffer system	supporting electrolyte
100 mM phosphate/Tris buffer	15 mM MgCl_2_ + 5 mM NaCl
1× TAE buffer	15 mM MgCl_2_ + 5 mM NaCl

Measurement buffers were produced at 0.2 pH intervals within the range, to electrochemically observe a switching point and switching dynamics of the zipper. Each pH buffer condition was spiked with either 2 mM Fe(CN)_6_^(−3/–4)^ in 100 mM KCl^–^, to give a working concentration of either 200 μM or 500 μM Fe(CN)_6_^(−3/–4)^.

#### 2.2.4 Electrode Functionalization

After cleaning, electrodes were immersed in ethanol for 3 min, rinsed in DI-H_2_O, and then dried under a steady argon stream. Electrodes were functionalized by overnight incubation (18 h) at 37 °C, in a solution of thiolated DNA origami at a concentration of 1 nM with backfilling agent MCP (3-mercapto-1-propanol), with an excess of 10 fold origami, all in the presence of an excess of the reducing agent TCEP (tris(2-carboxyethyl)phosphine hydrochloride) (10 μM). For the immobilization of a particular structural conformation, appropriate pH conditions are essential. Therefore, electrode functionalization is undertaken using a buffer of the necessary pH as the solvent within which DNA origami and MCP are diluted. This ensures conformity in the layers produced and provides necessary confidence in the starting conformation of the structures prior to any measurements.

Following this step, electrodes are named as functionalized electrodes (FEs). This coimmobilization protocol of introducing DNA structure and backfilling agent to the electrode at the same time has been previously identified as a simple and reliable method of establishing functionalized electrodes.

#### 2.2.5 Sample Characterization

Following overnight incubation, an initial determination of FE layer characterization was undertaken. FEs were allowed to incubate in the relevant buffer containing a spiked volume of redox mediator for a minimum of 15 min prior to initial measurement. This duration was chosen to help prevent signal drift due to fluid mechanical effects on the monolayers associated with the introduction of new buffers. If electrodes were ever subject to a buffer switch, this 15 min incubation was deemed necessary to negate the most severe incidence of signal drift. This incubation period is also sufficient to allow migration of ferri/ferrocyanide ions into the layer. During buffer switching, electrodes were rinsed in the deionized water for 10 s.

#### 2.2.6 Electrochemical Measurements

Electrochemical measurements were undertaken in a conventional three-electrode cell (working PGE, platinum counter, and saturated Ag/AgCl^–^ reference). An Autolab PGSTAT302N potentiostat (Metrohm-Autolab, Utrecht, Netherlands) was employed to run all measurements. An electrochemical script was written to characterize surfaces via differential pulse voltammetry (DPV) (potential window −0.1 to 1.6 V, step 5 mV), square wave voltammetry (SWV) (potential window −0.1 to 1.6 V, frequency 50 Hz, step 5 mV), and electrochemical impedance spectroscopy (EIS). The EIS response was measured at a frequency range of 10 kHz to 0.1 Hz, and the associated spectra were fitted to a simplified Randles circuit (Supporting Information Figure S6), with the *x*^2^ value determining the goodness of fit.

## 3 Results and Discussion

### 3.1 Characterization of the DNA Zipper Structure

For pH sensing, the modular DNA zipper (Figure 1) was functionalized with nine copies of pH locks. The active, pH-sensitive zippers were designed with nine copies of 18-nt long Hoogsteen-type DNA triplexes with a %TAT = 66.7 for an approximate p*K*_a_ of 7.6.^32,47^ For the open controls, the ssDNA counterparts of the triplexes were substituted with scrambled DNA sequences that cannot take part in triplex formation (the sequences for the active zippers and the control zippers are presented in Supporting Information Figure S1). According to an AGE analysis, both types of zippers were folded successfully and they could be efficiently purified from excess staples with PEG precipitation. They also remain intact in pH 6.5 and pH 8.0 TAE buffers and in the pH 6.5 phosphate buffer (Supporting Information Figure S2).

**Figure 2:**
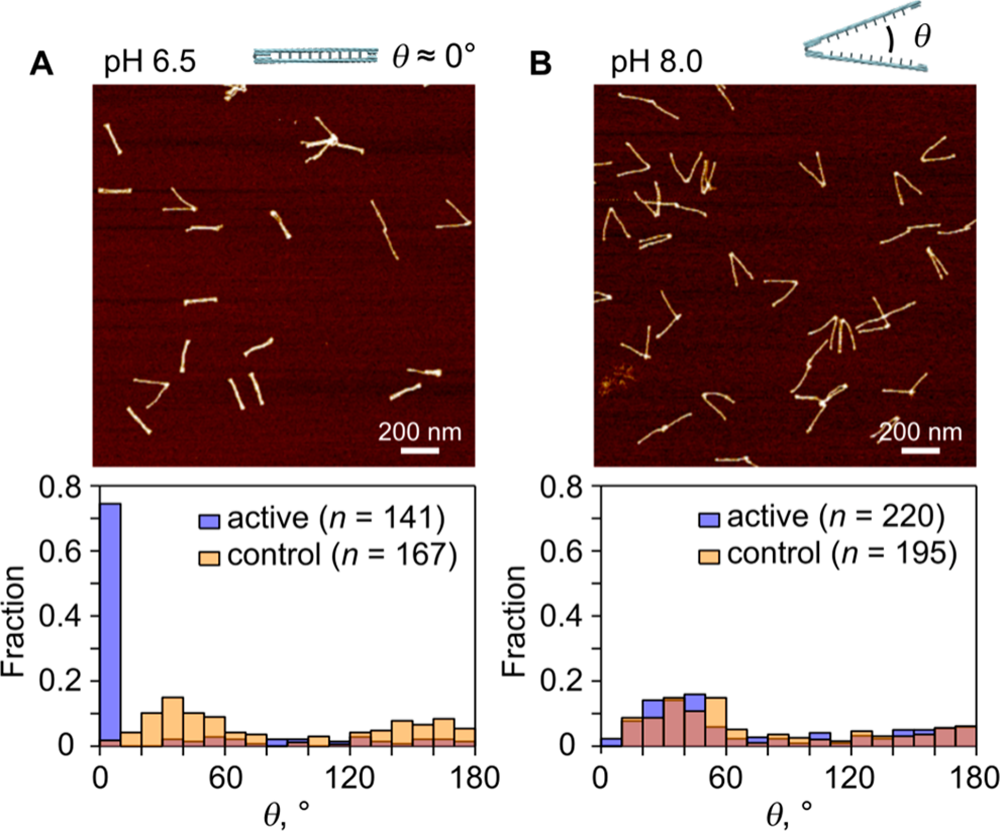
AFM analysis of the zipper conformation in TAE buffers. (A) AFM image of the active zippers at pH 6.5 (top panel) and the distribution of vertex angles (θ) measured for both the active zippers and the open controls. *n* denotes the number of individual structures analyzed for each sample type. (B) Active zippers at pH 8.0 (top) and statistics of the vertex angles of active and control zippers. Larger area AFM images and images of the open control zippers are presented in Supporting Information Figures S3 and S4.

The pH functionality of the DNA zippers was first confirmed with AFM imaging after incubating the samples overnight either in a pH 6.5 or in a pH 8.0 TAE buffer supplemented with 15 mM MgCl_2_ and 5 mM NaCl. At pH 6.5, the pH-responsive zippers were predominantly in a tightly closed conformation (Figure 2A). On the basis of an image analysis of the opening angles of the immobilized zippers, ∼74% of the pH-responsive zippers at pH 6.5 displayed a vertex angle of 0–10° corresponding to a closed configuration. At pH 8.0, the active zippers were in an open configuration and a wide distribution of vertex angles was observed (Figure 2B). The appearance of the active zippers in the open state was similar to the open controls at both pH 6.5 and pH 8.0. The result shows that the buffer pH induces a significant conformational change and a closing of the active zippers specifically due to the triplex formation, while the open controls stay in the open configuration at both pH values. Furthermore, only ∼2% of the active zippers at pH 8.0 and open controls at pH 6.5 were fully closed, showing that the closed conformation is highly unfavorable unless stabilized by a triplex formation.

In addition to zippers with a closed configuration, the active zipper sample incubated at pH 6.5 was observed to contain some amount of agglomerated structures (Supporting Information Figure S3). The low pH did not induce agglomeration of the open controls (Supporting Information Figure S4). This shows that the aggregation takes place in solution when the zippers are able to form contacts with each other through formation of DNA triplexes between individual structures. The agglomerates disassemble fast after the solution pH is increased, as indicated by an AGE analysis where no aggregation of the pH 6.5 TAE samples is observed on a pH 8.3 gel (Supporting Information Figure S2). The functionality of the active zippers in pH 6.5 phosphate buffer containing 15 mM MgCl_2_ and 5 mM NaCl was also studied. Closed and structurally intact zippers were seen in the AFM imaging, but both AFM and AGE analysis suggested a larger extent of agglomeration than in pH 6.5 TAE (Supporting Information Figures S2 and S5). Further, to assess immobilization of structures through the gold–sulfur bond, additional imaging of the thiolated-DNA zippers was carried out using gold substrates prepared by PVD (Supporting Information Figure S8). This was sufficient to illustrate the successful immobilization, and the minimal incidence of structural agglomeration. Therefore, this provides a confirmatory assessment of zipper immobilization. However, resolution of zipper structural conformation is enhanced with a mica substrate and was therefore chosen as an optimum surface for defining its vertex angle distributions at different pHs.

### 3.2 Electrode Functionalization

**Figure 3:**
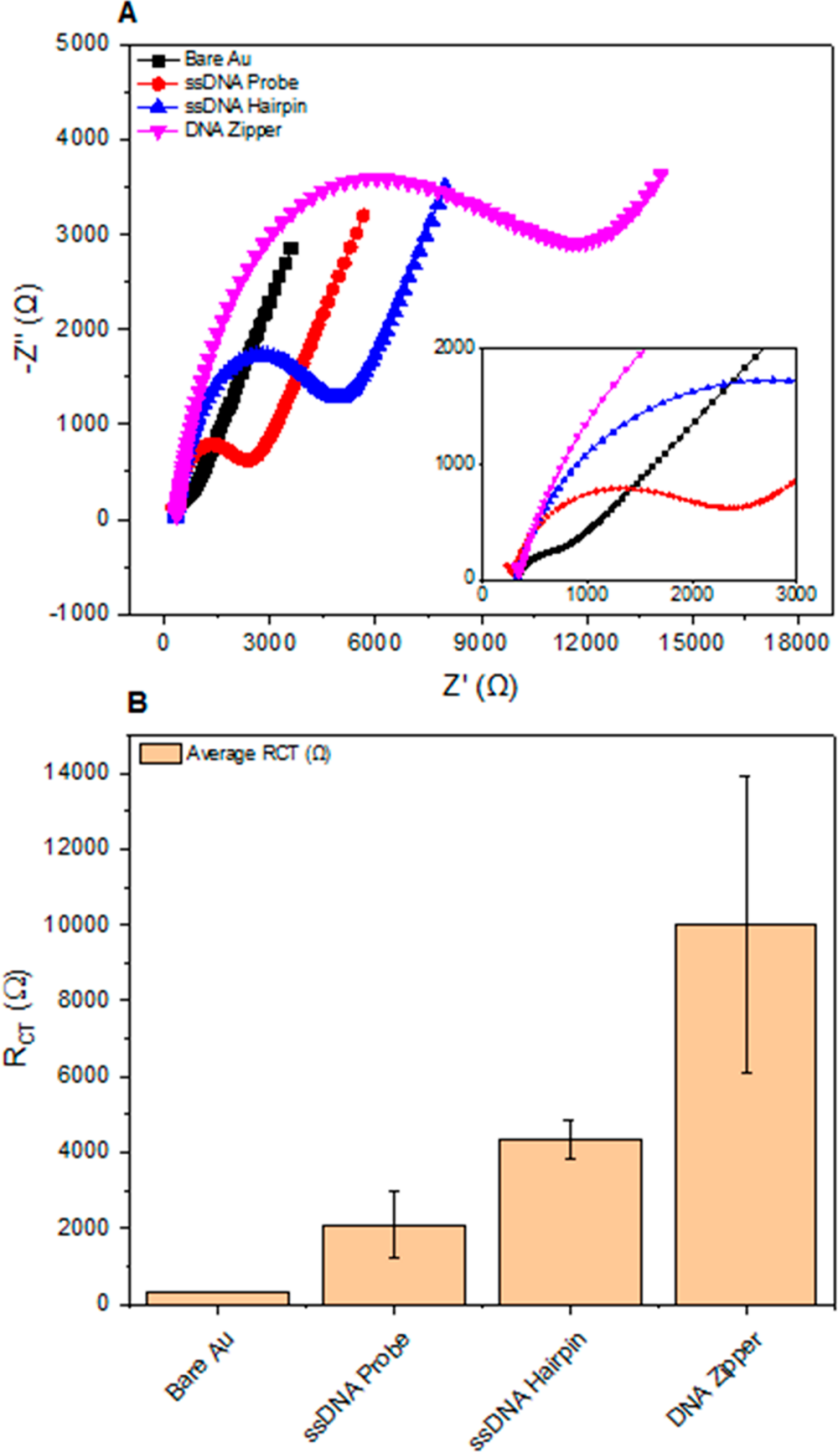
Electrochemical assessment of zipper immobilization on PGE. (A) Averaged Nyquist plots (inset: Nyquist responses at the high frequency range). (B) Comparison of averaged *R*_CT_ (Ω) for bare gold and various DNA SAMs (20 nt ssDNA probe and 91 nt ssDNA hairpin); 2 mM Fe(CN)_6_^(−3/–4)^ in 100 mM KCl^–^; *n* = 4 PGE per condition.

Having designed and produced the thiolated DNA zipper structure, it was then necessary to characterize its resultant immobilization characteristics on gold electrode surfaces. In this study, polycrystalline gold electrodes were selected because of the ability to clean in piranha solution (to remove organic contaminants) and to regenerate these surfaces with high repeatability using standard electrode polishing techniques. To assess the immobilization behavior of the DNA zipper, an experiment was carried out where both differential pulse voltammetry (DPV) and electrochemical impedance spectroscopy (EIS) at open circuit potential were performed in potassium ferri/ferrocyanide solutions to assess comparative surface functionalization. Potassium ferri/ferrocyanide (Fe(CN)_6_^(−3/–4)^) is a commonly employed redox couple for the measurement of DNA immobilization on electrode surfaces. The ferri- and ferrocyanide species possess trivalent and quadrivalent anions, meaning that interaction with immobilized DNA (a polyanion) is governed by electrostatic repulsion at an electrode surface. Comparisons of surface characteristics are drawn between the immobilized zipper, an immobilized DNA hairpin structure, an immobilized single stranded DNA probe, and a pristine electrode surface. EIS is a sensitive and label-free method for probing interfacial parameters, obtaining kinetic information, and monitoring mass transport-limited processes at modified electrode surfaces. In this technique, a small AC potential signal is applied at the working electrode and the resulting current response is measured. This is performed over a range of frequencies and allows parameters such as the solution resistance (*R*_S_), the double layer capacitance (*C*_DL_), and the charge transfer resistance (*R*_CT_) to be extracted. Figure 3 shows the EIS results from electrode functionalization experiments by contrasting the zipper’s behavior with the immobilization characteristics of a linear ssDNA probe (20 nt) and a ssDNA hairpin structure (91 nt).

Figure 3A shows typical Nyquist plots and a good representation of the impact of the zipper’s large size (∼4.7 MDa) following surface functionalization, by comparison with simple DNA films (hairpin and liner probe) associated with common biosensor designs. It can be seen in Figure 3A that despite the concentration of zipper being 10 nM in comparison to the 1 μM concentrations of ssDNA probe, and ssDNA hairpin immobilization solutions, the value of charge transfer resistance increased by ∼130% compared to that of the ssDNA hairpin. Here, measurement of the zipper was undertaken in 2 mM Fe(CN)_6_^(−3/-4)^ in 100 mM KCl^–^ buffer, which is in keeping with a common electrochemical buffer principle employed in DNA biosensing work. Note that the pH of the measurement buffer at this point has not yet been established, and specific structural conformation is not clear. Compared to the ssDNA probe and ssDNA hairpin structure, variation of zipper states may account for the high variation associated with zipper *R*_CT_ values displayed in Figure 3B which is a bar chart with error bars summarizing impedimetric responses of the different modified electrode surfaces. Having successfully confirmed zipper immobilization by EIS, it was necessary to determine the minimum concentration of redox mediator, Fe(CN)_6_^(−3/-4)^, required to allow effective signal transduction through the DNA zipper-containing film on the electrode surface. Previous studies have noted potential drawbacks to the use of higher concentrations of ferri/ferrocyanide with gold substrates, primarily from cyanide ion damage to the gold surface and resultant signal drift.^52,53^ A Fe(CN)_6_^(−3/–4)^ buffer at 500 μM was sufficient to resolve consistent DPV traces in the μA range, with oxidation peaks occurring at ∼200 mV (see Supporting Information Figure S7).

### 3.3 Investigating pH-Induced Conformational Switching of the DNA Zipper

To determine the validity of the hypothesis that a change in the electrochemical signal could be associated with the pH-driven opening of the zipper, a control structure was introduced into this study. The control structure had no pH locks within the flexible arms of the zipper, and as such the molecule could not adopt a closed conformation. Alongside comparative measurements between the active, pH-responsive zipper and the control structure, the importance of the buffer system and its background contribution to signal changes was investigated. Comparisons were drawn between the ability of each buffer to resolve the structural conformation. Phosphate/Tris and TAE buffer systems were chosen for their appropriate buffering capabilities across the pH range under investigation.

**Figure 4:**
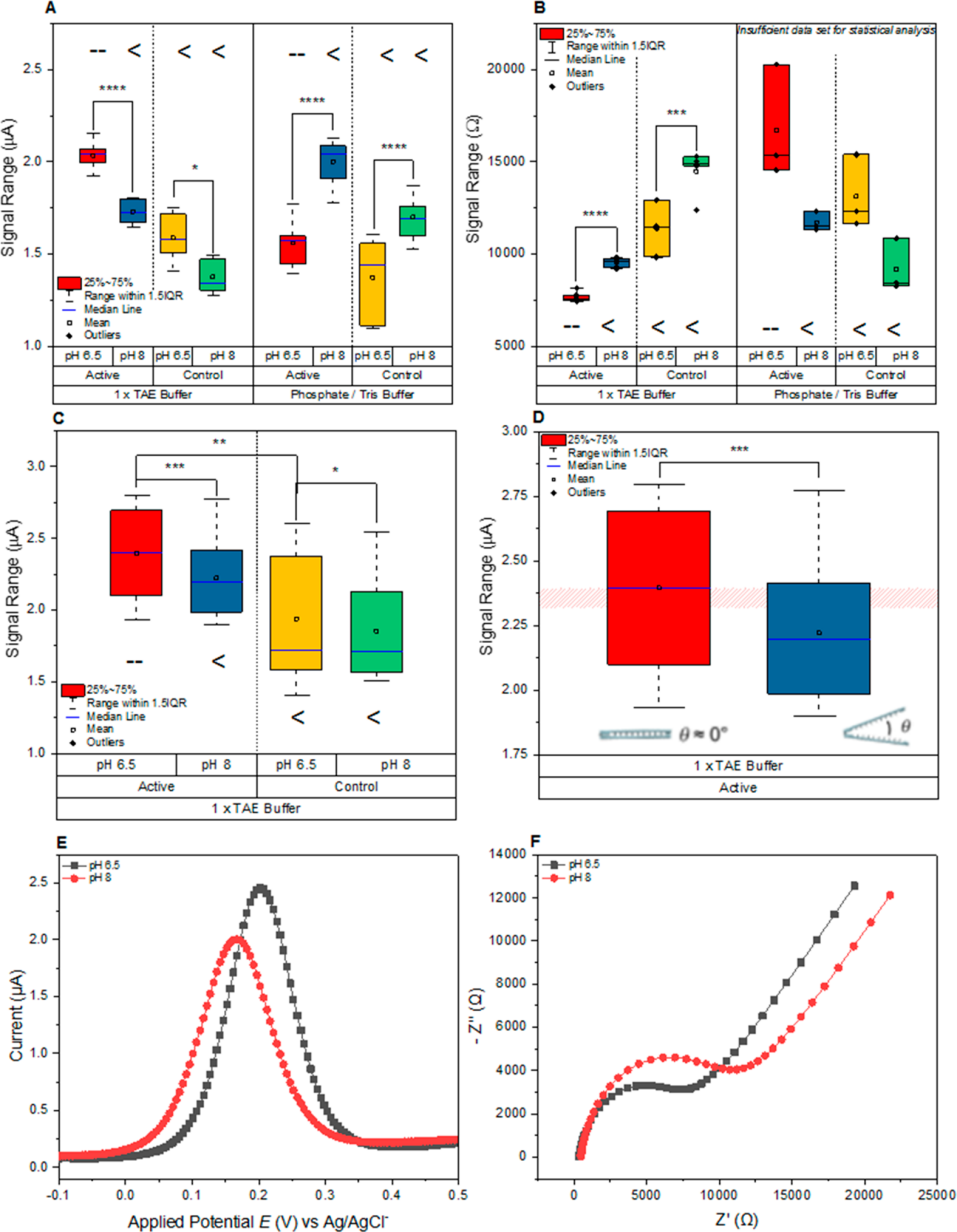
Peak current data of active pH-responsive zipper and control open zipper following immobilization on PGE in a closed starting conformation and representative DPV and Nyquist responses of the active zipper. (A) Box plot of peak currents (μA). (B) Box plot of charge transfer resistance (*R*_CT_) (Ω). (C) Peak current data (μA) of 1× TAE buffer measurements, following the subtraction of signal drift associated with electrochemical behavior for each pH state. (D) Peak current data (μA) of 1× TAE buffer measurements, for active zipper (red bar = pH 6.5, blue bar = pH 8). Pink band represents threshold signal change required to exceed the contribution from a yet unknown parameter which is present in the control panel of (C). (E, F) Representative DPV and Nyquist responses of active zipper to buffer pH 6.5 and 8, respectively. (Levels of significance given at ns *p* > 0.05, *p ⩽ 0.05, **p ⩽ 0.01, ***p ⩽ 0.001, ****p ⩽ 0.0001). (A) and ((C) and (D)): *n* = 4 and *n* = 12 PGE, respectively, with triplicate measurement per condition. (B) *n* = 4 PGE for 1× TAE system, 3 PGE for phosphate/Tris buffer system, single measurements for EIS.

Figure 4 shows the results from a series of experiments designed to understand changes in the electrochemical signal for two pH values, in different buffer systems by contrasting the responses of active and control zippers (Figure A–D shows peak current data of active pH-responsive zipper and control open zipper on PGE in a closed starting conformation, and Figure 4E,F the representative DPV and Nyquist responses of the active zipper).

In Figure 4A, the switching of pH contributes to a highly significant increase in observed DPV peak current for both active, pH-responsive zippers and control zipper-modified electrodes when supported by a phosphate/Tris buffer system (*p* < 0.0001 for both). AFM/PAGE data (Supporting Information Figure S5) support the evidence provided here of the phosphate/Tris buffer system being suboptimal, with reduced substrate coverage and yield. We hypothesize that signal change is a combination of two factors. First, poor film formation on the electrode surface and its subsequent reorganization, and second, the altered electrochemical behavior exhibited by Fe(CN)_6_^(−3/–4)^ when the electrodes were exchanged between phosphate and Tris buffer solutions. By employing a 1× TAE system, which appears preferential in the origami synthesis process, it is easier to resolve peak current variation associated with the opening of the zipper structure (*p* < 0.0001 and *p* = 0.0236 for active and control, respectively). While this is an improvement, the signal change in our active system cannot yet be conclusively attributed to a switching event alone.

Mean charge transfer resistance as presented in Figure 4B, for both the active and control zipper structures in the 1× TAE system, was subject to highly significant increases in signal following a pH change, with *p* < 0.0001 and *p* = 0.0003, respectively, between the open and closed states. Sensitivity of this measurement technique may play some role in this, with the incidence and severity of layer reorganization, or nanoscale pinhole effects, being substantially amplified. Despite this, one order of magnitude exists between the significance of active and control responses, further hinting at a contribution from opening zippers on the electrode surface.

In its closed conformation, the phosphate-rich backbone of the DNA zipper means that the structure bears a high negative charge density and strong electrostatic barrier, localized around the closed zipper structures. The relative surface coverage of the zippers is low, and we hypothesize that the backfilling agent MCP, at a concentration 10 times that of the zipper, predominates across large areas of the surface, thus leading to a surface with distinct regions of discrete negative charge. Previous works have noted that mixed films of 1 μM ssDNA and mercaptohexanol (MCH) at a 1:1000 ratio can harbor 10^12^–10^13^ DNA strands per cm^2^.^54,55^ With a low concentration, the impedance of the layer is predominantly a function of the large size and significant negative charge density. Ultimately, further work is required to determine the true surface coverage of the zipper, and chronocoulometry approaches like those developed by Steel et al.^56^ may provide a quantitative assessment.

The use of trivalent and quadrivalent anions of the ferri- and ferrocyanide species enable probing of the changes to the electrostatic repulsion from the polyanionic DNA zipper structures in their open and closed configurations. Remembering that the zippers appear to be present on the surface as discrete entities, we hypothesize that in the closed conformation, this electrostatic repulsion of the redox mediator is limited to only the environment proximal to an immobilized zipper. Upon opening, the flexible arms of the zipper separate from one another and position themselves out into solution. The impact of this is a decrease in the density of charge around the zipper structures but development of a more diffuse negatively charged barrier extending further out across the electrode surface and into solution. This in effect serves to produce a greater barrier to electron transfer between Fe(CN)_6_^(−3/–4)^ and the underlying gold substrate, which manifests as an increase in charge transfer resistance (Figure 4B) and decrease in DPV peak current (Figure 4A and 4C).

Supporting Information Figure S7 highlights the impact of buffer pH on basic electrochemical measurements with pristine unmodified gold electrodes. The DPV signal change associated with this pH switch in 1× TAE with 500 μM Fe(CN)_6_^(−3/–4)^ and 100 mM KCl^–^, from 6.5 to 8, equates to a decrease of approximately 227 nA or 7.03% in peak current. It is therefore necessary to account for this phenomenon through subtraction of the artifact from our experimental data set, which is presented in Figure 4C. This yields an overall reduction in the level of significance, for signal decreases associated with both the active and control zipper (*p* = 0.0004 and *p* = 0.0487, respectively). We can therefore hypothesize that there is a yet unexplained phenomenon contributing to redox currents in both active and control experiments. However, it cannot be the sole cause of signal changes associated with the active zipper. Comparison between the data sets of active and control structures at pH 6.5 yields a highly significant difference in mean peak current (μA), indicating that the active zipper is in fact being immobilized in a closed conformation, prior to it opening with the introduction of an alkaline buffer.

Finally, a threshold signal change has been determined in Figure 4D, with the pink band representing the % change (−3.27%) of mean peak current (μA) observed in the control panel. Here our measured signal change in the active zipper exists outside this band, with a peak current reduction of 7.05%, or 173.6 nA. We have now accounted for two contributing factors influencing peak current: first, the known impact pH has on the electrochemical behavior of our redox couple Fe(CN)_6_^(−3/–4)^, and second, the influence of an additional parameter that is well observed but yet to be conclusively defined. Figure 4E and 4F shows real DPV and Nyquist signal response to changing buffer pH, with a reduction in peak current and gain in *R*_CT_, respectively, as pH shifts from 6.5 to 8.

AFM images presented in Supporting Information Figure S3 highlight the incidence of structure agglomeration unique to zippers in their closed conformation. It is possible that the protocol for immobilization of DNA zippers presented in this paper yields islands of agglomerated structures on the electrode. Signal change associated with the switching of buffer pH from acidic to alkaline may have a contribution from the opening of the zipper leading to a breakup of these clusters and a film reorganization. Work is currently ongoing to determine the incidence of agglomeration in our system and the contribution that breakup of these masses may provide to the overall signal change.

In totality, the results shown in Figure 4 clearly demonstrate that once baseline effects and measurement artifacts were removed, it was possible to probe the conformational states of the zipper structure within different pH regimes using label-free electrochemical methods. The interrogation of the control zipper side by side with the active structure gives great confidence that the conformation can be switched over the two pH values, and this can be resolved through EIS and DPV measurements. These experiments show that the electrochemical signal can be representative of the zipper conformation opening up several sensing applications including pH probing. The zipper could be potentially deployed on its own in a calibrated system or in an array-based system alongside the control structure to give a differential measurement which in effect removes all background effects and signal artifacts.

## 4 Conclusions

This study introduces a pH-responsive thiolated DNA zipper capable of adopting closed and open configurations at pH 6.5 and 8.0, respectively. By immobilizing the structure onto gold electrode surfaces and removing background artifacts arising from altering the buffer conditions, it was possible to reliably discriminate between the closed and open configurations of the zipper in two different pH regimes (6.5 and 8.0) using simple, label-free electrochemical measurements. These findings provide a platform for future developments which include addition of secondary functions to these structures, including biorecognition elements for sensing applications, release of relevant cargo molecules upon opening, or direct sensing of pH in complex media such as blood.

## Supporting Information Available

Additional figures and tables as noted in the text (PDF)
