# Supplementary material for: Probing the Conformational States of a pH-Sensitive DNA Origami Zipper via Label-Free Electrochemical Methods
Source: Langmuir. 2021 Jun 15;37(25):7801–9. doi: 10.1021/acs.langmuir.1c01110 (PMC8280702; doi:10.1021/acs.langmuir.1c01110)
Supplement: Supplementary file 1 — la1c01110_si_001.pdf [file la1c01110_si_001.pdf]

Supporting Information

Probing the Conformational States of a pH-Sensitive DNA Origami Zipper *via* Label-Free Electrochemical Methods

Paul Williamson,[†,⊥] *Heini Ijäs,*[‡,§,⊥] *Boxuan Shen,*[‡] *Damion K. Corrigan,**[,†] *and Veikko Linko**[,‡,//]

[†] Department of Biomedical Engineering, University of Strathclyde, 40 George Street, Glasgow G1 1QE, United Kingdom

[‡] Biohybrid Materials, Department of Bioproducts and Biosystems, Aalto University, P.O. Box 16100, 00076 Aalto, Finland

[§] Nanoscience Center, Department of Biological and Environmental Science, University of Jyväskylä, P.O. Box 35, 40014 Jyväskylä, Finland

[//] HYBER Centre, Department of Applied Physics, Aalto University, P.O. Box 15100, 00076 Aalto, Finland

*Corresponding authors: Email: damion.corrigan@strath.ac.uk; Email: veikko.linko@aalto.fi

List of supplementary figures and tables

Figure S1. The zipper structure and the sequences of the pH lock residues.

Figure S2. Agarose gel electrophoresis (AGE) analysis of the zippers after folding, purification, and buffer exchange.

Figure S3. Additional AFM images of the active zippers in TAE buffers.

Figure S4. AFM images of the control zippers in TAE buffers.

Figure S5. AFM images of the active zippers in pH 6.5 phosphate buffer.

Figure S6. Simplified Randles Circuit for the fitting of Electrochemical Impedance Spectroscopy data.

Figure S7. Impact of Redox Mediator $Fe(CN)_6^{(-3/-4)}$ concentration, pH on redox mediator performance.

Figure S8. AFM images of the thiol-modified zippers immobilized on a gold surface.

Tables S1–S3. Staple strand sequences for the DNA zippers.

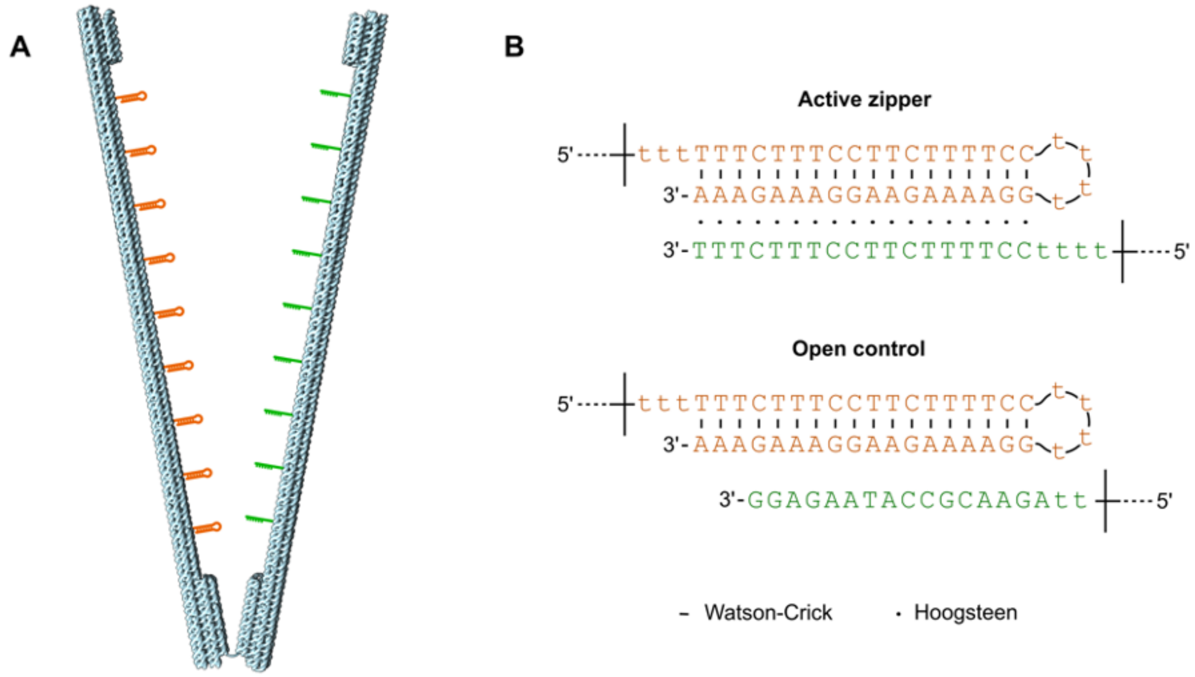

Figure S1. The zipper structure and the sequences of the pH lock residues. (**A**) Illustration of the DNA zipper structure, showing the location of the pH lock residues. (**B**) Sequences of the DNA hairpin- and ssDNA staple strand extensions in the active zippers (top panel) and in the open controls (bottom panel), with an illustration of the Watson-Crick and Hoogsteen hydrogen bonds.

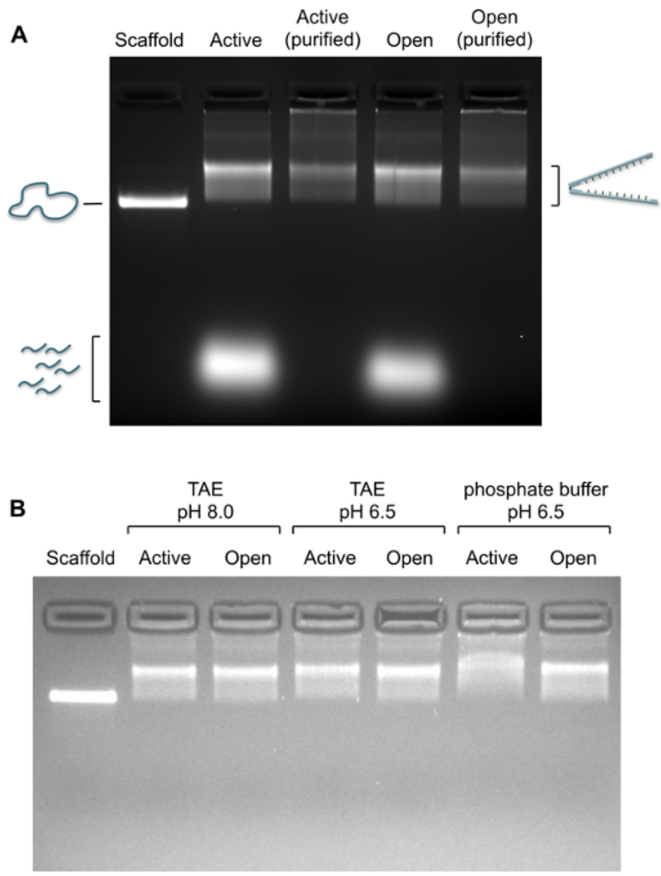

Figure S2. Agarose gel electrophoresis (AGE) analysis of the zippers after folding, purification, and buffer exchange. (**A**) Comparison of the electrophoretic mobility of the 7,560-nt DNA scaffold (lane 1), assembled zippers in the presence of excess staple strands (lanes 2 and 4), and PEG-purified zippers (lanes 3 and 5). (**B**) The electrophoretic mobility of the 7,560-nt scaffold (lane 1) and PEG-purified zippers that have been incubated in different buffer conditions before the gel run. The high pH of the gel (~8.3) can be expected to convert all structures into an open configuration and lead to a disassembly of aggregates. The slower migration of structures in pH 6.5 phosphate buffer may originate from the better buffering capacity of the phosphate buffer at pH 6.5 preventing pH-induced changes in the sample.

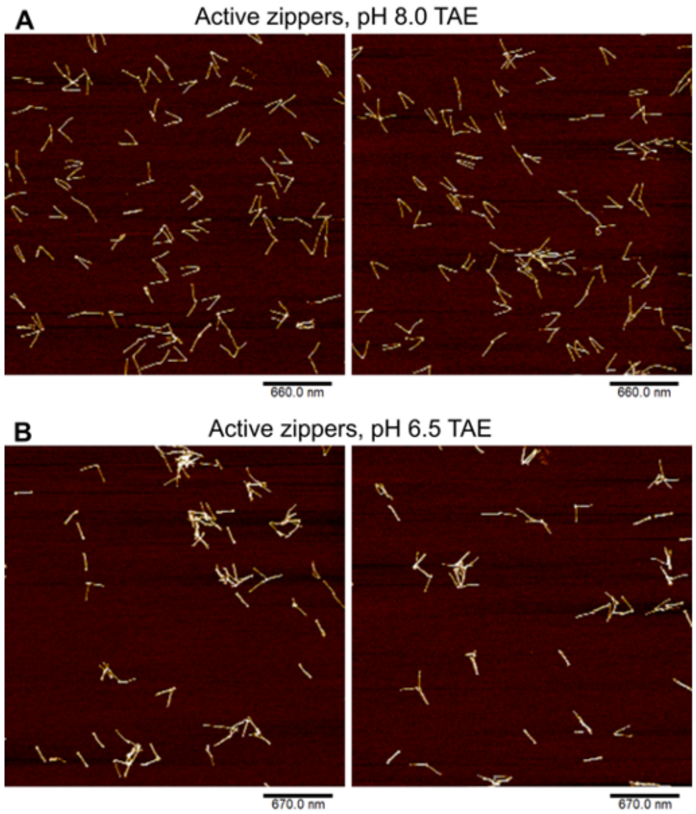

Figure S3. Additional AFM images of the active zippers in TAE buffers. (**A**) pH 8.0. (**B**) pH 6.5.

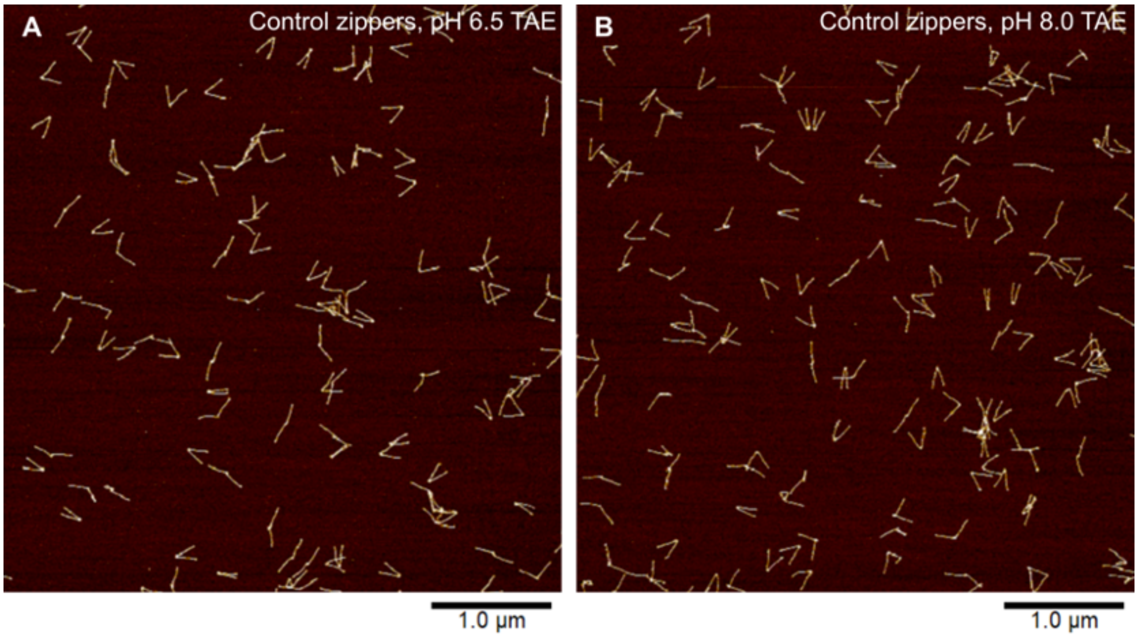

Figure S4. AFM images of the control zippers in TAE buffers. (**A**) pH 6.5. (**B**) pH 8.0.

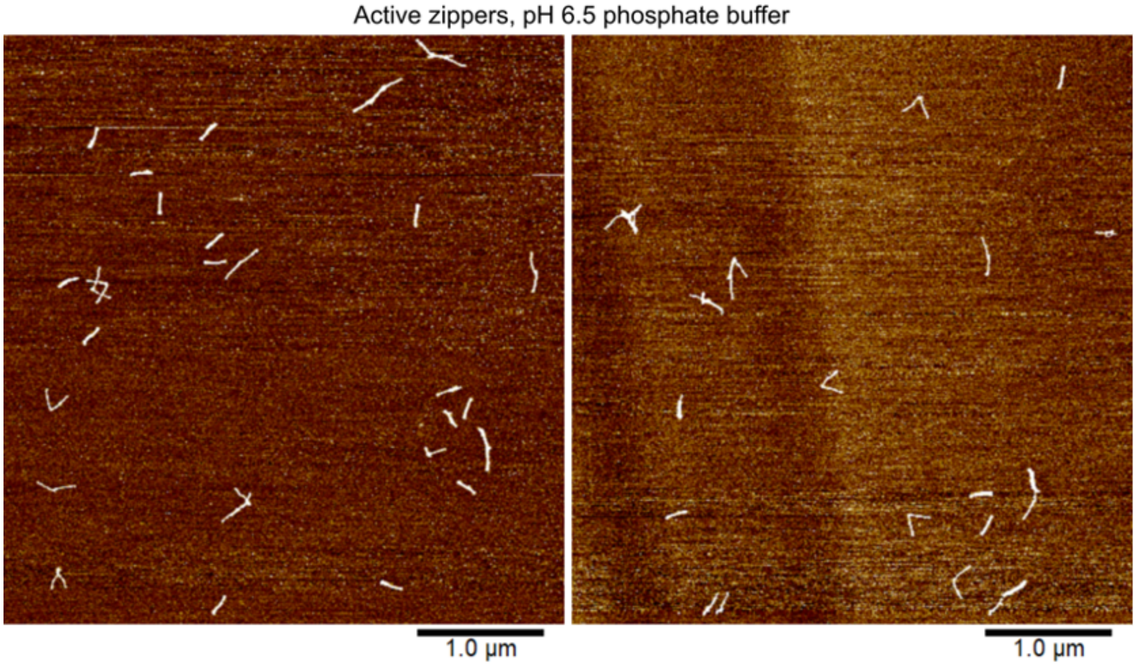

Figure S5. AFM images of the active zippers in pH 6.5 phosphate buffer.

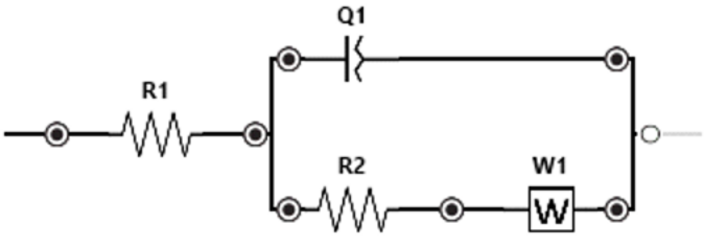

Figure S6. Simplified Randles Circuit for the fitting of Electrochemical Impedance Spectroscopy data, and subsequent determination of charge transfer resistance (Rct) (Ω). R1 is solution resistance, R2 is charge transfer resistance, Q1 is double-layer capacitance modelled by an imperfect capacitor, W1 is the impedance associated with bulk diffusion in solution (Warburg Element).

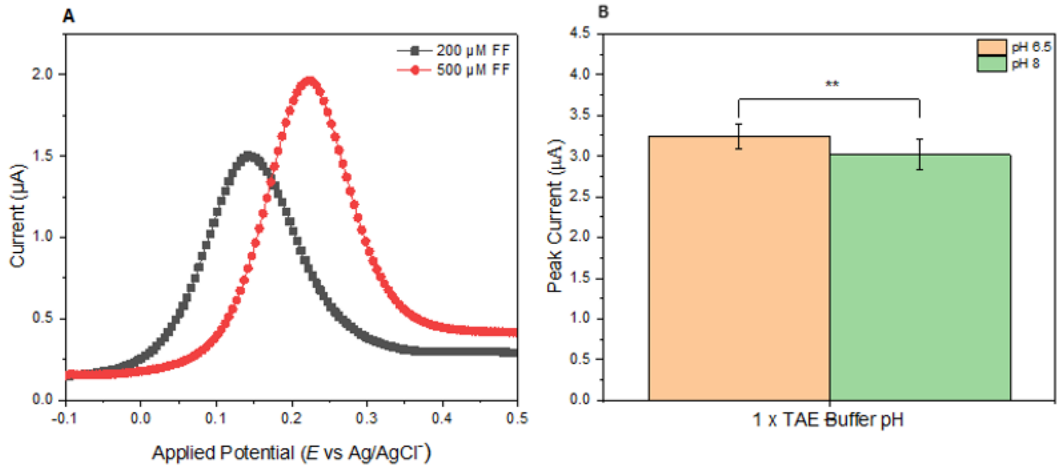

Figure S7. (**A**) Typical DPV traces for two concentrations of $Fe(CN)_6^{(-3/-4)}$. (**B**) Impact of Changing Buffer pH on the Basic Electrochemical Behaviour of FeCN. DPV peak current (µA) of FF at pH 6.5 and 8 on pristine gold electrodes. p = 0.0093, with a % reduction of 7.03, or 227.6 nA.

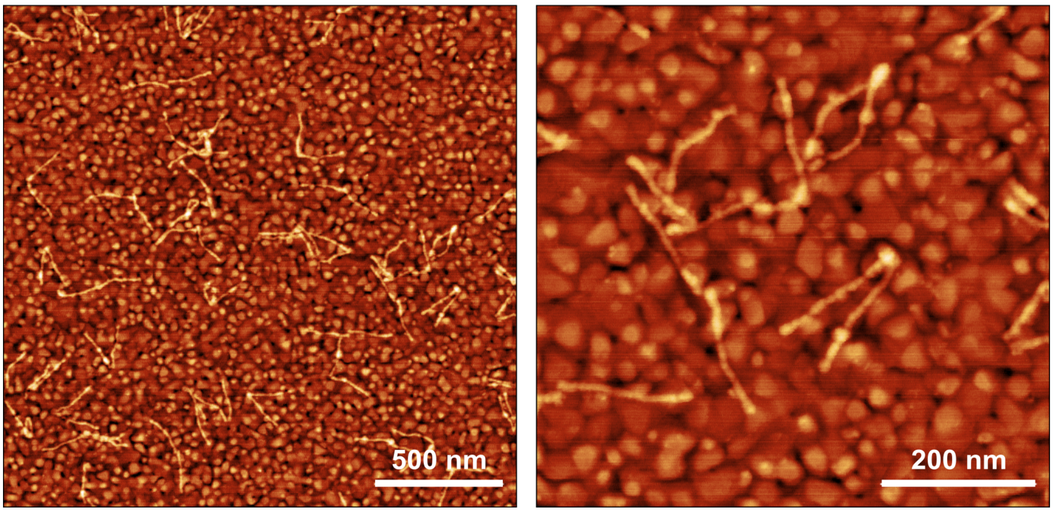

Figure S8. AFM images of the thiol-modified zippers immobilized on a gold surface (2 nm Ti + 20 nm of Au). The surface was prepared using physical vapor deposition.

Table S1. Staple strand sequences for folding the core zipper structure without pH lock extensions nor strands for immobilization. For folding the active zippers or the open controls, the indicated strands for the modification sites A - I (green and orange; site A is located closest to the hinge of the zippers and site I furthest from the hinge) should be replaced with corresponding extended strands in Table S2. For immobilization, the indicated core strands (purple) are replaced with the thiol-modified stands in Table S3. Unpaired poly-T sequences (poly-T overhangs used for passivating the helix ends and poly-T spacer sequences in strand extensions) are written in lowercase letters.

Arm 1		
**Strand no.**	**Sequence**	**Strand type**
[1]	GAGCTTGGTGTAGCAGTCTCTTATTTTG	core
[2]	CGAACGTCGGGCGCAGTAAGCCCTTAAATCAAGATCGGTATTGTCAGAT	core
[3]	TTCTGACATTCTGGAAATTGTTAGCAAACGTAGAGAATAAATTTCAT	core
[4]	TTAATGCGCGAAAGTTGAATGGCTATTAATATGGTTTTTG	core
[5]	CTAAAGGTAACCACTAAAGCCTTTATCC	core
[6]	GGCCCACCCTCAAGTTAACGG	core
[7]	GGCACAGACGACAAAATAAA	core
[8]	CCCAAATAAGTATTGTAACAG	core
[9]	GGTCGAGGTACCTATTGTTAATG	core
[10]	GGGCTGCGCGGAGCCCCTTTGG	core
[11]	GATTGTTCGCCTGATCGTAGGAATCATTTACCAACAAATAAA	core
[12]	ATTCCTGGCGAATTGTACCGCACTCATCTTACAAAAGGTTGA	core
[13]	GCGGAACAAACAAAGGCTGTCTTTCCTTGAAACGAGCCACCA	core
[14]	GAACGTTTCATTTGTCCCATCCTAATTTTTTACAGACCCTCA	core
[15]	ACTTTACATATATGTGTTTATCAACAATGGGAGAAGGAACCA	core
[16]	AGCCGTCTTAATTACGACGACAATAAACTGAGCGCCCCCTTA	core
[17]	TATCTAACTTAGATGAATATAAAGTACCAGCCCAAGTCAGAC	core
[18]	GGTCAGTAAATCATACATGTAATTTAGGATCTTACAGCACCG	core
[19]	CACCTTGGGTTGGGACAGTAGGGCTTAAAACAAAGATTAGCA	core
[20]	TGCCACGATCGCAATATGCGTTATACAAGGAATACTTGGGAA	core

[21]	GAACCCCTAAATTAAGGCGTTAAATAAAAATACCAAAGA	core
[22]	GCTTTTGCAAAAGAAATCCAAAAAAAAAGCGCC	core
[23]	CGCGTACTACAGACGAGCCTAATTTGCCAGGAGAACAAAAAT	core
[24]	CGAGCACGTTGACAGGATAAACAGCCATATAAACCAAATTCA	core
[25]	CCTCGTTAGCACCAGACCCAATCCAAATAAATCATTCAAAAG	core
[26]	AGCTAAACACAGAGCCTTTTTTGTTTAACGAATAATCCATCA	core
[27]	AAGGGATTTCACCCTCTGAAAATAGCAGCCACGAGCATACAT	core
[28]	GTACGCCAGAGCCGCCAGAGAATAACATAAAATAATAAATTA	core
[29]	TGTTTTTATCACCGGAGAAGCGCATTAGACAGATAAGCAGTA	core
[30]	CACCGAGTAAATCACCTTAACTGAACACCCACGCGCCTGAGT	core
[31]	CATCACGCATTGCCATAGTCAGAGGGTAATAACATGTGTAAA	core
[32]	TAGCAATACTCATAGCTAATATCAGAGAGAGTCCAGAATTTT	core
[33]	TAATAACATCGTTTTCCAAGAATTGAGTTAGACAAAAAAACA	core
[34]	TAGAAGAACCTTTAGCTAATAAGAGCAAGAAATAAGATAAGA	core
[35]	CCTTGCTGGTAGCGACAAATAGCAATAGCTCAGAGGCTAGTG	core
[36]	CAATATTACCGATAGCCGAAGCCCTTTTTAAACGCCAAGGTC	core
[37]	CAACAGGAAAAACGTCTAAGCAGATAGCCGTTGAGAATAACC	core
[38]	AAATACCTACATTACCTTACCAGAAGGAAAACGCTCATTATA	core
[39]	CAATCGTCTCAGCAAAAACGCAATAATAACATTCTTACTGAT	core
[40]	TTACATTGGGAGCCATCCAAAAGAACTGGCAGTATCAGACAA	core
[41]	TCACACGACAATTATCAGACTCCTTATTACATTACTATTTTCAA	core
[42]	TTAATGCCACAAACGCTAACGAGCGTCTATTTTCATTGCT	core
[43]	CGTTTTTTTCCAGATTGGCCTTGATATTGCCGCTATAATCCT	core
[44]	GGGTATTTATTTATGCCGCCGCCAGCATATAACGTTCATCAT	core
[45]	ACCAATCTCAAAAAAGAGCCACCACCCTGGAGGCCTCATTTT	core
[46]	CAAGAAAAAACAGGACCGCCTCCCTCAGAATCCTGTTTGCCC	core
[47]	ATGCAGATGAACAACTTTTCATAATCAAAAAGAGTGTATTAG	core

[48]	TAATTCTTAACCCAATCGGCATTTTCGGTTCTTTGAGATTAG	core
[49]	CGCGAGGTTTGAAGGTCATACATAAGTTAGAAGGA	core
[50]	AGCCAGTAACAATGAGAATCAAGTTTGCTCAAACTGGAAGGT	core
[51]	ATTTAACAGAAAAGACCAATGAAACCATCGCCAGCAATATCT	core
[52]	AAAGCCACCGAGGAATCACCAGTAGCACCATTTTGAAAGCAT	core
[53]	CCTGTTTATGATTAACCGTCACCGACTTCAGATTCGCAACAG	core
[54]	AATCATAGCAGTATATTCATTGGGAAGGGGGCGAC	core
[55]	GCTTATCTAGTTGCGAATTTAGCCTTGAAAGAGGC	core
[56]	CAAGCAACTACAATAGAATGGATAAACAATTCTGA	core
[57]	TTCAGAACAGAAAAAGGAGGGCGAGAGGGCGAT	core
[58]	GAATATAATCAAAACTGGCAAACGGGGAACCATCA	core
[59]	TTGAATACCATCAATACAGGG	core
[60]	TTTCAATTAGGAATTAGCTTT	core
[61]	AGAAAACAAAACATTAGATTA	core
[62]	CCTTTTTTATAAATCCAGAAG	core
[63]	GAATAACCTTTTAGAACTGTC	core
[64]	CCCTTAGAAAACTAATATTAG	core
[65]	CGCTGAGAAGAATTGAATCGG	core
[66]	TGAGAGACTCTCAATCCATTG	core
[67]	TAACTATATAAAATCTACGCT	core
[68]	AGAACGCGAACCGCCTACCAG	core
[69]	ATATATTGGTGAGGAAAAGGGACCTGAATACGT	core
[70]	CTTCTGAACCAGCAAGCTGATAGCCCTAAGAAACGATACA	core
[71]	GGCAGGTTGGTTGCGATGATGGCAATTCAAGTTACAGCAAGC	core
[72]	GAACCACAATCAGACCACCAGAAGGAGCCCTGAGCCAAGAAC	core
[73]	GAACCGCTAGACAGTTAAAAGTTTGAGTAATTAATTGTAGAA	core
[74]	GAGCCACAATCAGTTCGACAACTCGTATATGGAAATCCTGAA	core

[75]	TTAGCGTAATTAACAATACATTTGAGGATGCTTCTTCAGCTA	core
[76]	TGTAGCGCACTTGCTTAGGAGCACTAACTCCTTGAGGTAAAG	core
[77]	TAATCAGTAATATCTCAACAGTTGAAAGGAGTCAAATTTTCG	core
[78]	AGGCCGGAAACGCTTCAAATATCAAACCACCTTTTTCGCCAT	core
[79]	TTAGAGCGAAATGGCCAGCAGCAAATGAGTAAATGCCAGTAT	core
[80]	AAAGGTGCAGTAATCGGTCAGTATTAACGAAAACTGAAAAAG	core
[81]	CACCACACGGCCAACGAAGATAAAACAGATTAGTTAACACCGG	core
[82]	CACCCAGATCAGATAGTACCTTTTACATGGAA	core
[83]	TGAATCTACCGCGCAACAATAACGGATTTGGATTAGCCGCGC	core
[84]	GCCAAAAATATTTATAGAACCC	core
[85]	AACATGACAAGTTTCGATTTA	core
[86]	TTTCGGAGCCGTAACGGAACC	core
[87]	GGTCAGTCCGTTCCTAGGGCGTTATTTGCACGTAAGTTTAACCTAAGAA	core
[88]	TGCCCGTAAAGCGCGGTCACGTTAGAACCTACCATCAGTAACATAGAAG	core
[89]	CCCCCTGTCCTCATCACACCCTACTTCTGAATAATCGGGAGACCAATAG	core
[90]	TATTGGGAATAAGTTTATTACCAGC	core
[91]	ttttttttAATACCGAAC	core + poly-T
[92]	ttttttttAGAAAGCGATAAAGAAtttttttt	core + poly-T
[93]	ttttttttGTGATAAATTAATGGTtttttttt	core + poly-T
[94]	ttttttttTGCGTAGATT	core + poly-T
[95]	ttttttttCATATAAAAAACATCGtttttttt	core + poly-T
[96]	ttttttttCGGGAGGTCGTTTTAGtttttttt	core + poly-T
[97]	TAAAGGTGGCtttttttt	core + poly-T
[98]	ttttttttTACTGGTAATGGCTTTtttttttt	core + poly-T
[99]	TCACAATCAAtttttttt	core + poly-T
[100]	AAAAACACTGCCGCCACAGGCGGAtttttttttttttttTGAGACTTACGTGAAAGCCGG	core (staple connection to arm 2)
[101]	CGCGCAGAGATTATCATTTGA	core (modification site A)

[102]	AAGATGATGAAAGAAAGCGGG	core (modification site B)
[103]	TTAACAATTATTAATTGAACG	core (modification site C)
[104]	CATAAATCAAAACAATGAGGC	core (modification site D)
[105]	TCGTCGCTAAATAGATCGTTG	core (modification site E)
[106]	TAGCGATAGAATATCTCTGAG	core (modification site F)
[107]	AATTTATCATGGCAAACAGAA	core (modification site G)
[108]	TCCGGCTTACTGAACCCATGG	core (modification site H)
[109]	GCAAATCCACTGAGAGATTAT	core (modification site I)

Arm 2

Strand no.	Sequence	Strand type
[110]	GCCACCAAACCCATGTCCACGTTGTTAA	core
[111]	GAACAGGGTTGATATAAGGGA	core
[112]	GCCACCCTACAACGATTGCCCAAAATAA	core
[113]	GAGGCAAAAGAATTACGTAAT	core
[114]	TTTTGCTAAGGGCGTCCTGTT	core
[115]	TAAGTGCAGAACGTAAATCGG	core
[116]	TAAACGGGAGTGACCGACAGTGCGGCCCTGCCAATTTACGCT	core
[117]	GCCACTAAATCTATGTCCCGCCAAAATATGTCAACCTCATCTAAAGTAC	core
[118]	ATTCATTCCAGGAGGTTCGGAATA	core
[119]	ATTATTACCTCAAATGTCAATCATATGTTTCATCAGTTTTTC	core
[120]	GGAAGAAAATCAAAAGCAAACAAGAGAACTCCGTGAACGCGC	core
[121]	TGCGATTCAAAGCGAGAGATCTACAAAGCACGTTGAGTCGGG	core
[122]	GAGATGGACTTCAATTCTAGCTGATAAAGTTTGAGGCTAACT	core
[123]	TGCCCTGGACCGGAGGCCGGAGACAGTCCTCCAGCCCGGAAG	core

[124]	CCAAATCTTAATTGTGCCTGAGTAATGTGGCAAAGTGTGAAA	core
[125]	CTTCATCTAGAGCTAAGGATAAAAATTTAGGGCGATACCGAG	core
[126]	ACAGATGTTAAATAGACCCTGTAATACTGAAAGGGACGTGGT	core
[127]	GGTCAATATATAACCCTCAGAGCATAAAAGGGTTTCTGACCT	core
[128]	CGCGACCTTTGACCCATACAGGCAAGGCCAAGCTTTGAATCG	core
[129]	AACGGAGTAGCTATCATCAATTCTACTAGGTTGTGCATCTGT	core
[130]	AAAACACTTATGAAATTCATGCGCACGAAGGTGGATTTT	core
[131]	CAGACAGCAGTGAGTCCTGTAGCCAGCTACCCCGGATTCATTGAA	core
[132]	TCGTAAACGAGTAATGCGTATTGGGCGCTCTAAAGGAACAAC	core
[133]	GCCTGAGATTGACCAGCTGCATTAATGATCTGTATGACGTTG	core
[134]	GGTAGCTATCGTAATGCGCTCACTGCCCGAGTGAGTACCTTA	core
[135]	ATGATATATCGGCCCCTGGGGTGCCTAAAATTTTTTGGGCTT	core
[136]	AAAAGGGCGCTTCTCAATTCCACACAACGGAGCCTTAAGGCT	core
[137]	TATTTTACAGGCTGCATGGTCATAGCTGGAATTTCTCATTAC	core
[138]	CTTTATTTTCGCTATAAAGACGGAGGATGACAACAGGCTGAC	core
[139]	ACCAAAAGCGATTATGAGTAAACAGGGCTGAGGCTAAAGAGG	core
[140]	ATTAAGCTTGTAAACATAAATCATTTCTACCCTCACGCAGAC	core
[141]	TTAAATTCATTTTTAGTTGCAGGTTCCGGGACTCC	core
[142]	AACATCCAGGGTGGTGGGCACGAATATAGGCTACACGAAATC	core
[143]	GAAGATTGCCATCATTCACCGCCTTATAGAGTCCA	core
[144]	TCAGAAACTGGCCTACGGGCACCCGAGATGTTGTT	core
[145]	TCCCCAGGTAGAGTTAGCGTAACGACAGG	core
[146]	CATAAAATCTATAGTAAATGAATTTATCG	core
[147]	GAAGTTAAGAACAACAGTTTCAGCGGCTT	core
[148]	AAAGTTTAATTGAATTGCGAATAATTGAG	core
[149]	ACCAACGAGAAAAAAGGCTCCAAAAATAC	core
[150]	ACCTAACGTAACTTGCTTTCGAGGTTTTC	core

[151]	GGCTAAGAGTAGTTGCGCCGACAATCCCC	core
[152]	TGTTAACGGTGATATATTCGGTCGCTTAA	core
[153]	TTCCCATAAGGTTTGCGGGATCGTCCCGA	core
[154]	TTAGTGCTCCAACGAGGGTAGCAACGGGG	core
[155]	ACATTTCATTGTGTGAGGCTT	core
[156]	GTGACATTAAATGTGAGACTAGCATGCTTTAAAC	core
[157]	GTTCAACCCGTCGGATTTCGATGACGAGAATGAC	core
[158]	GGGGAGACAGACGTCGTTAATAAAACGACAGAAAAACGGTAA	core
[159]	GCCGGAACAAACGGCGGAGTCTGGAATCAGGTCT	core
[160]	GCCGTAATGGGATAGGTGCTATCAATTATAGTCA	core
[161]	AAACCTGCAACTTTCTGGCTCATTATACCCTGACTGGTCATT	core
[162]	TCCGTGTAGATGGGCGCATTTTTGGATTGCATCA	core
[163]	CGTCCGTGCATCTGCCATTAATGCAGGAAGCCCG	core
[164]	CACATTAACTAAAGTCAACTTTAATCATGATTAAGCGGAGAG	core
[165]	TGAGGGACGACGACAGTTCAACCGATATCGCGTT	core
[166]	AAGTCAGGAAGATCGCAAAATCACTTCAAAGCGA	core
[167]	CATAAAGTCCAAAAACACCAGAACGAGTTTCGAGCCATCAAT	core
[168]	GAGCAGCTTTCCGGCACTGAGAAAAGCAAACTCC	core
[169]	TCAGGTGCCGGAAACCAGTAGGTAATTAGAGAGT	core
[170]	TTGTTATTTATCAGCAAAGCTGCTCATTGGTCAGGAAGATTC	core
[171]	CTGCGCCATTCGCCATTAATGCAACTCCTTTTGA	core
[172]	AATCGCAACTGTTGGGATTAGAACTTTTGCGGAT	core
[173]	CTCGAATACCGATAATCTTGACAAGAACAGGTCATCCTCATA	core
[174]	GGGTCGGTGCGGGCCTCTCAACGCTAATTGCTGA	core
[175]	CGATTACGCCAGCTGGCTTTGCGGTAGCTCAACA	core
[176]	GCTTGTTATAACCGTACAGACCAGGCGCAATGCTGGAGAAGC	core
[177]	GCTGGATGTGCTGCAAGACATTATTGCAACTAAA	core

[178]	TAAAGTTGGGTAACGCCGCTAAATGGAAGTTTCA	core
[179]	CCTGGTTGGCCGCTGAACCGAACTGACCGGTGTCTCGGTTGT	core
[180]	ACTTCCCAGTCACGACGAATAAAGAGTTGATTCC	core
[181]	TCAACGACGGCCAGTGCAAAGAATACGAGTAGAT	core
[182]	GCTGACGCATCGGATGTTACTTAGCCGGTCTGCGATAGCAAA	core
[183]	CCTTCTCAGGAGAAGCCAATAAATATTAGAT	core
[184]	AAGCAACTAAAGACTCATCGCCTGATAAGCAAATGTAGCATT	core
[185]	ATCAGCTGTAAACGCGACGATATAGCGAG	core
[186]	TCTAAGTATAGTAGGTCAATAACCTGTTATTTGTATTTACACT	core
[187]	TAGGAACGTATAAGTTTAGACTGGATAGTCAACTATACAAACTCA	core
[188]	TTCGCGTAGCCCCATACTGCGGAATCGTTGAG	core
[189]	AACGTCACAGTACCCCTCAGA	core
[190]	CGCCCTGGTAAAATTCATGAGGAAGTTTTGAGGACTCGTCGGATGTTCT	core
[191]	CGCTTCTCGAAGGCAACGAAA	core
[192]	CTATTAACGTCGAGCGCCACCCTCAGAAAGTTTCGCTGAGAGTAACCAA	core
[193]	ACAAAATCAAAAGAATAGACAGCTGCCTGTAGTAGGAAT	core
[194]	CCAGTTTTATAGCCTAGTACC	core
[195]	TGATGGTGCAAGCGGTACCGTAGGAATTACGAGGCTACCAGATTAAT	core
[196]	CAAAATCCCTGGCCTCACCAGATGCAGA	core
[197]	TTTTCACCCCTCATAAAGATT	core
[198]	ttttttttCGCGAAACTTGACCCCtttttttt	core + poly-T
[199]	ttttttttCCAATAGGCCCTCATTtttttttt	core + poly-T
[200]	ATTTTGTTAAtttttttt	core + poly-T
[201]	ttttttttGAGCTGAAACTTAAGTtttttttt	core + poly-T
[202]	ttttttttCCCTCGTTATAGTAAGtttttttt	core + poly-T
[203]	CATTTGGGGCtttttttt	core + poly-T
[204]	ttttttttAGTGCTGAAT	core + poly-T

[205]	tttttttTTAAATTTCTGGTTTGttttttt	core + poly-T
[206]	ACCACATCGTCCAAAAAACAG	core (thiol)
[207]	CATCAGTCATAAATTTGATAA	core (thiol)
[208]	TACATAATAAAATGCAAATAT	core (thiol)
[209]	AGTTACTAACGTTTTGTCGTCTTTCGGCG	core (modification site A)
[210]	TTACCAGTCAGGGGATTTTGCTAAATCGT	core (modification site B)
[211]	AAAATGTGAATAATAGAAAGGAACAATTG	core (modification site C)
[212]	TTAAAGTAAATTCACGTTGAAAATCTGTA	core (modification site D)
[213]	AACACAGTGAATTAATTGTATCGGTCCGC	core (modification site E)
[214]	TAAGCGGATATTTAAACAGCTTGATTCGT	core (modification site F)
[215]	ATATATAGGCTACCATCGCCCACGCACCT	core (modification site G)
[216]	GTACAACTTTGTGCAGGGAGTTAAAGGTG	core (modification site H)
[217]	CAATAACGAGGGCAGCGAAAGACAGCATT	core (modification site I)

Table S2. Extended staples for the hairpins and ssDNA counterparts (Figure S1) of active and control zippers.

Arm 1: DNA hairpins for both active and control zippers		
**Strand no.**	**Sequence**	**Strand type**
[101]	CGCGCAGAGATTATCATTTGAtttTTTCTTTCCTTCTTTTCCttttGGAAAAGAAGGAAAGAAA	core + 3' hairpin (modification site A)
[102]	AAGATGATGAAAGAAAGCGGGtttTTTCTTTCCTTCTTTTCCttttGGAAAAGAAGGAAAGAAA	core + 3' hairpin (modification site B)
[103]	TTAACAATTATTAATTGAACGtttTTTCTTTCCTTCTTTTCCttttGGAAAAGAAGGAAAGAAA	core + 3' hairpin (modification site C)

[104]	CATAAATCAAAACAATGAGGCtttTTTCTTTCCTTCTTTTCCttttGGAAAAGAAGGAAAGAAA	core + 3' hairpin (modification site D)
[105]	TCGTCGCTAAATAGATCGTTGtttTTTCTTTCCTTCTTTTCCttttGGAAAAGAAGGAAAGAAA	core + 3' hairpin (modification site E)
[106]	TAGCGATAGAATATCTCTGAGtttTTTCTTTCCTTCTTTTCCttttGGAAAAGAAGGAAAGAAA	core + 3' hairpin (modification site F)
[107]	AATTTATCATGGCAAACAGAAtttTTTCTTTCCTTCTTTTCCttttGGAAAAGAAGGAAAGAAA	core + 3' hairpin (modification site G)
[108]	TCCGGCTTACTGAACCCATGGtttTTTCTTTCCTTCTTTTCCttttGGAAAAGAAGGAAAGAAA	core + 3' hairpin (modification site H)
[109]	GCAAATCCACTGAGAGATTATtttTTTCTTTCCTTCTTTTCCttttGGAAAAGAAGGAAAGAAA	core + 3' hairpin (modification site I)

Arm 2: ssDNA extensions for active zippers

Strand no.	Sequence	Strand type
[209]	AGTTACTAACGTTTTGTCGTCTTTCGGCGttttCCTTTTCTTCCTTTCTTT	core + pH lock extension (modification site A)
[210]	TTACCAGTCAGGGGATTTTGCTAAATCGTttttCCTTTTCTTCCTTTCTTT	core + pH lock extension (modification site B)
[211]	AAAATGTGAATAATAGAAAGGAACAATTGttttCCTTTTCTTCCTTTCTTT	core + pH lock extension (modification site C)
[212]	TTAAAGTAAATTCACGTTGAAAATCTGTAttttCCTTTTCTTCCTTTCTTT	core + pH lock extension (modification site D)

[213]	AACACAGTGAATTAATTGTATCGGTCCGCttttCCTTTTCTTCCTTTCTTT	core + pH lock extension (modification site E)
[214]	TAAGCGGATATTTAAACAGCTTGATTCGTttttCCTTTTCTTCCTTTCTTT	core + pH lock extension (modification site F)
[215]	ATATATAGGCTACCATCGCCCACGCACCTttttCCTTTTCTTCCTTTCTTT	core + pH lock extension (modification site G)
[216]	GTACAACTTTGTGCAGGGAGTTAAAGGTGttttCCTTTTCTTCCTTTCTTT	core + pH lock extension (modification site H)
[217]	CAATAACGAGGGCAGCGAAAGACAGCATTttttCCTTTTCTTCCTTTCTTT	core + pH lock extension (modification site I)

Arm 2: ssDNA extensions for control zippers

Strand no.	Sequence	Strand type
[209]	AGTTACTAACGTTTTGTCGTCTTTCGGCGttAGAACGCCATAAGAGG	core + control extension (modification site A)
[210]	TTACCAGTCAGGGGATTTTGCTAAATCGTttAGAACGCCATAAGAGG	core + control extension (modification site B)
[211]	AAAATGTGAATAATAGAAAGGAACAATTGttAGAACGCCATAAGAGG	core + control extension (modification site C)
[212]	TTAAAGTAAATTCACGTTGAAAATCTGTAttAGAACGCCATAAGAGG	core + control extension (modification site D)

[213]	AACACAGTGAATTAATTGTATCGGTCCGCttAGAACGCCATAAGAGG	core + control extension (modification site E)
[214]	TAAGCGGATATTTAAACAGCTTGATTCGTttAGAACGCCATAAGAGG	core + control extension (modification site F)
[215]	ATATATAGGCTACCATCGCCCACGCACCTttAGAACGCCATAAGAGG	core + control extension (modification site G)
[216]	GTACAACTTTGTGCAGGGAGTTAAAGGTGttAGAACGCCATAAGAGG	core + control extension (modification site H)
[217]	CAATAACGAGGGCAGCGAAAGACAGCATTttAGAACGCCATAAGAGG	core + control extension (modification site I)

Table S3. Thiol-modified strands for arm 2.

Arm 2		
**Strand no.**	**Sequence**	**Strand type**
[206]	/5ThioMC6-D/tttttACCACATCGTCCAAAAAACAG	thiol modification + core
[207]	/5ThioMC6-D/tttttCATCAGTCATAAATTTGATAA	thiol modification + core
[208]	/5ThioMC6-D/tttttTACATAATAAAATGCAAATAT	thiol modification + core
